# Genetic and Clinical Spectrum of Hereditary Transthyretin Amyloidosis in Brazil

**DOI:** 10.1111/jns.70097

**Published:** 2026-01-07

**Authors:** Gustavo Maximiano‐Alves, Carolina Lavigne‐Moreira, Marcus Vinicius Simões, Adilson Junior Pinto Galvão, Flavio Henrique Valicelli, Fernando Saraiva Coneglian, Elisa Vegezzi, Pedro Manoel Marques Garibaldi, Pedro José Tomaselli, Andrea Cortese, Wilson Marques

**Affiliations:** ^1^ Department of Neuroscience and Behavioral Sciences, Ribeirão Preto Medical School University of São Paulo São Paulo Brazil; ^2^ Department of Neuromuscular Diseases UCL Queen Square Institute of Neurology London UK; ^3^ Cardiology Division, Department of Internal Medicine, Ribeirão Preto Medical School University of São Paulo São Paulo Brazil; ^4^ Multidisciplinary Center for Amyloidosis/Neurocop—Irece Salvador Brazil; ^5^ IRCCS Mondino Foundation Pavia Italy; ^6^ Department of Medical Imaging, Hematology, and Clinical Oncology, Ribeirão Preto Medical School University of São Paulo São Paulo Brazil; ^7^ National Institute of Sciences and Technology—INCT‐Translational Medicine—CNPq/FAPESP São Paulo Brazil

**Keywords:** age of onset, genetic variability, hereditary transthyretin amyloidosis, peripheral neuropathy, presymptomatic screening

## Abstract

**Background:**

Transthyretin hereditary amyloidosis (ATTRv) clinical variability has been widely reported, not only across countries and variants but also among families and distinct regions within a single nation. One of the principal challenges in disease management is the accurate determination of age of onset (AOO), which is heterogeneous and has therapeutic implications given the availability of disease‐modifying treatments.

**Methods:**

This study characterizes the genetic landscape and clinical onset spectrum of ATTRv in an admixed Brazilian cohort of 175 patients.

**Results:**

Seven *TTR* pathogenic variants (p.Val50Met, p.Val142Ile, p.Ile127Val, p.Ile88Leu, p.Ala39Asp, p.Phe84Leu, p.Tyr98Phe) were identified. The most common was p.Val50Met (58.8%), followed by p.Val142Ile (29.7%) and p.Ile127Val (7.4%). Notably, 44% of V122I had a neurological onset. Close clinical monitoring of presymptomatic carriers reduced age at diagnosis by 10.5 years. The median AOO was 50 years, with V30M patients presenting earlier (38.5 years) than V122I (p.Val142Ile) (60y) and I107V (p.Ile127Val) (60 years). Familial cases showed a 20.5‐year earlier AOO than sporadic cases. In Brazil, late‐onset (> 50 years) V30M is more common than previously reported (37.5%); ethnicity can influence AOO within the same variant, and for the first time, we show a distinct geographic pattern: early‐onset V30M is more frequent in São Paulo/South, whereas late‐onset V30M predominates in the central region.

**Interpretation:**

This study emphasizes the heterogeneity of ATTRv presentation in admixed populations and underscores the need for expanded screening and multicenter studies to refine genotype–phenotypic correlations.

## 1 Introduction

Hereditary transthyretin amyloidosis (ATTRv) is a progressive and often fatal autosomal dominant disorder caused by the extracellular deposition of misfolded transthyretin (TTR) protein, predominantly affecting the peripheral nervous system and myocardium [1, 2]. Although over 120 *TTR* pathogenic variants have been identified globally [3], V30M (p.Val50Met) accounts for more than 50% of reported cases and is classically associated with a length‐dependent sensory‐motor‐autonomic polyneuropathy and/or restrictive cardiomyopathy [1, 2].

The clinical phenotype of ATTRv exhibits considerable heterogeneity, which is largely influenced by specific *TTR* variants. For instance, the V122I (p.Val142Ile) is predominantly associated with a cardiomyopathic presentation [4], I107V (p.Ile127Val) has been linked to cranial nerve involvement [5], and the S52P (p.Ser72Pro) is characterized by an especially aggressive disease course [6]. Nonetheless, there is also significant phenotypic variability in individuals carrying the same variant, and this phenomenon has been well documented in V30M (p.Val50Met) carriers. In endemic regions, such as Portugal, V30M (p.Val50Met) demonstrates high penetrance and is typically associated with early‐onset (< 50 years) small fiber neuropathy. Conversely, in Sweden and certain Japanese populations, the same variant exhibits reduced penetrance and manifests later in life, often as a large fiber neuropathy with symptom onset around 60 years of age [1, 2, 7].

Brazil is recognized as an endemic region of ATTRv, largely attributable to the Portuguese V30M (p.Val50Met) founder mutation, where the disease reportedly presents with a relatively homogeneous clinical phenotype of early‐onset small fiber neuropathy [8]. However, most available data derive from populations in Rio de Janeiro, where European ancestry predominates [8]. In contrast, regions with higher proportions of African and Indigenous ancestry, and with less common variants, remain underrepresented in the literature, raising the likelihood of underdiagnosis and limiting clinical characterization.

In this retrospective study, we sought to describe the clinical onset and genetic spectrum of Brazilian ATTRv patients of different ancestry, including European, African, East‐Asian, and *Pardo* (an official census category in Brazil referring to individuals of mixed European, African, and Indigenous heritage). The main goal is to describe the geographic distribution of the most common *TTR* variants, better characterizing their clinical variability and identifying factors possibly contributing to this heterogeneity in a highly admixed population.

## 2 Materials and Methods

### 2.1 Ethics

This study was approved by both local and national research ethics committees (CEP‐CONEP; CAAE: 74939423.4.0000.5440). Written informed consent was obtained from all participants prior to inclusion. All procedures were conducted in accordance with the ethical standards outlined in the Declaration of Helsinki and its subsequent revisions.

### 2.2 Clinical Data

Over 6 months (July 2024 to January 2025), data were retrospectively collected from medical records at the Peripheral Neuropathy section of a Multidisciplinary Center for Amyloidosis (MCIA), Hospital das Clínicas, Ribeirão Preto Medical School, University of São Paulo (HCRP‐FMRP‐USP). All patients harboring *TTR* pathogenic variants who agreed to take part in the study were enrolled during this timeframe. We focused on the age of onset, presenting symptoms, age at diagnosis, and epidemiological data that could possibly influence the age of onset. Other variables, such as severity scores, progression, and treatment were not included in this analysis.

As per clinical practice in our Institution, asymptomatic carriers are examined annually, while symptomatic patients are seen every 6 months. In these visits, every patient undergoes a structured neurological and cardiological evaluation. The neurophysiological protocol comprises a nerve conduction study, sympathetic skin response test [SSR], a thermal threshold Quantitative Sensory Test [QST] on the dorsal foot of one body side, electrochemical skin conductance test [Sudoscan], and Pain‐Related Evoked Potentials [PREP], all the reference values and protocol standardization for the tests were used as described in Papagianni [9]. The cardiological approach consists of blood tests (NT‐ProBNP and troponin), an electrocardiogram, echocardiography, and cardiac scintigraphy. In doubtful cases or inconclusive complementary tests, nerve, heart, abdominal fat, and skin biopsies were performed.

Small fiber neuropathy was determined as the presence of classical sensory or autonomic symptoms plus two abnormal neurophysiological tests or the presence of symptoms with an abnormal intra‐epidermal fiber density at skin biopsy, both associated with a normal nerve conduction study. Large fiber neuropathy was determined as the presence of sensory or motor symptoms plus altered physical examination or abnormal nerve conduction studies. We did not consider carpal tunnel syndrome when defining the age of neurological onset of the disease. Cardiac involvement was defined as cardiac scintigraphy showing increased cardiac uptake of technetium 99mTc pyrophosphate (PYP) (Perugini grade 2 or 3) or the presence of symptoms with interventricular septal thickness ≥ 12 mm at echocardiography and high levels of NT‐ProBNP and/or pacemaker implantation. The mere presence of symptoms with an abnormal electrocardiogram was not considered. Orthostatic hypotension was established as a ≥ 20‐mmHg decrease in systolic blood pressure or a ≥ 10‐mmHg decrease in diastolic blood pressure 3 min after standing up. Patients concomitantly with other etiologies of neuropathy, such as diabetes or leprosy, or other causes of cardiopathy, such as hypertension, were excluded to avoid possible confounders in the age of onset (Figure S1).

### 2.3 Genetic Test

*TTR* pathogenic variants were determined using Sanger sequencing. DNA was extracted from white blood cells using a commercial kit (Pure Gene, Gentra). All four *TTR* exons (ENST00000237014; NM_000371.4) were sequenced in both directions using the ABI Prism Big Dye Terminator cycle sequencing‐ready kit (Life Technologies), with electrophoresis and analysis using the software Sequencing Analysis Software 6 (Applied Biosystems) and 4Peaks (version 1.7.1).

### 2.4 Statistical Analysis

Data was stored using Microsoft Excel 365 Personal. Statistical tests were performed using IBM SPSS version 30.0. GraphPad Prism 10 was used for graphics design. Maps were created using Datawrapper (Datawrapper GmbH, Berlin, Germany). Data normality was assessed by the Shapiro–Wilk test. Descriptive statistics were presented as medians and compared with the Mann–Whitney and Kruskal–Wallis tests. Post hoc pairwise comparison was assessed by the Dunn–Bonferroni test.

We conducted a generalized linear model (regression analysis) to examine the association between type of variant, self‐reported ethnicity, presymptomatic clinical monitoring, and family history, with age of disease onset (years). Then we tested these predictors, adding place of birth (geographical region) to the V30M (p.Val50Met) subgroup. Multicollinearity was assessed using variance inflation factors (VIF). Associations between variables were examined using a chi‐square test of independence. All tests were considered statistically significant if the *p*‐values were lower than or equal to 0.05.

## 3 Results

### 3.1 Genetic Variability and Cohort Descriptions

**FIGURE 1:**
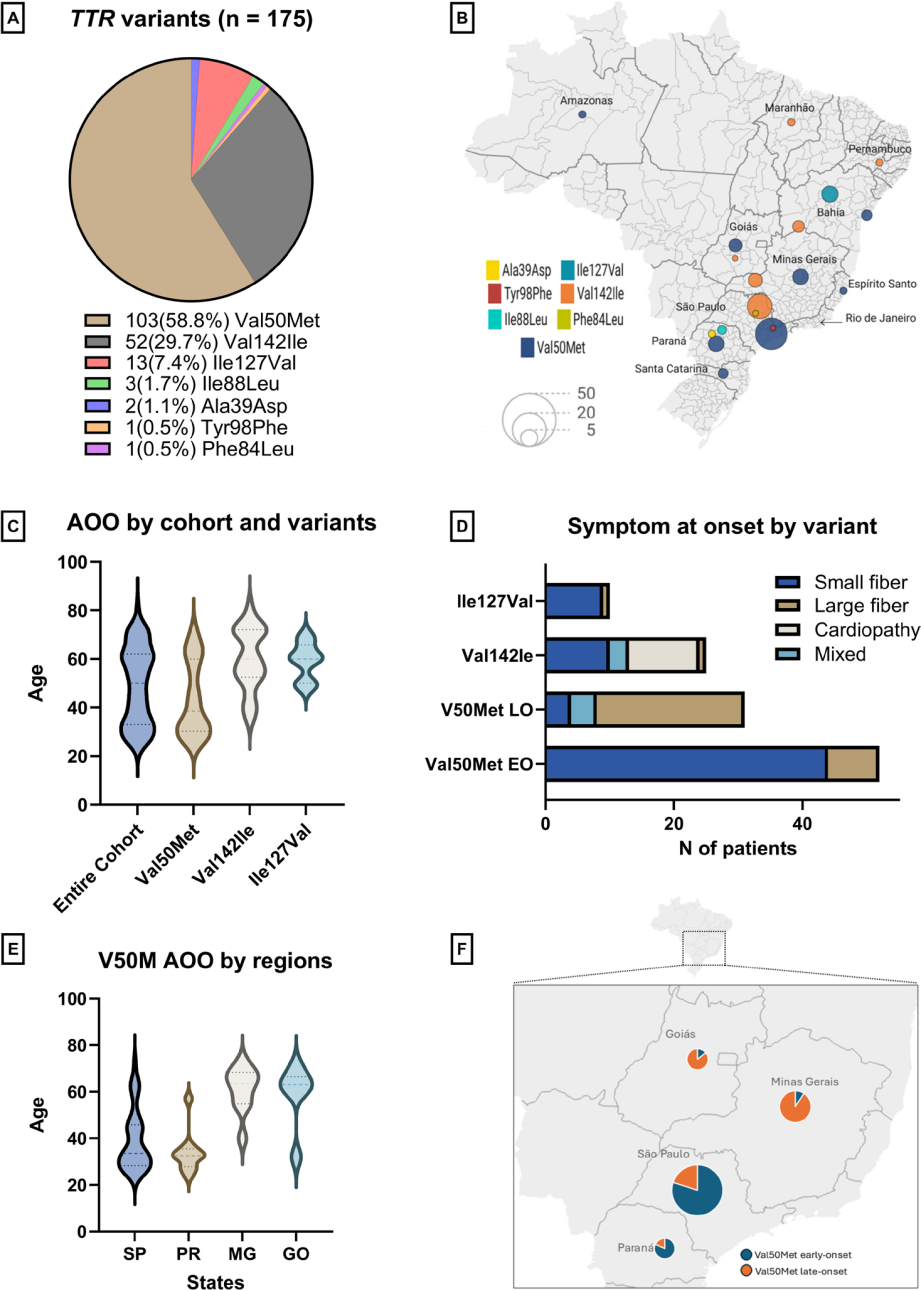
(A) *TTR* pathogenic variants identified in this study. (B) Brazilian map showing numerical and geographical distribution of variants. (C) Violin plots comparing AOO between the entire cohort and the three main variants. (D) Symptom at onset among the three main variants. (E) Violin plots showing the AOO among V30M (p.Val50Met) variant in the four main regions. (F) Inset shows a magnified view of the selected region (dashed box) from the main map. There is a predominance of V30M (p.Val50Met) early‐onset cases in São Paulo and Paraná (South) than in the Brazilian central region (Minas Gerais and Goiás). Maps were created using Datawrapper. AOO, age of onset; EO, early‐onset; GO, Goiás; LO, late‐onset; MG, Minas Gerais; PR, Paraná; SP, São Paulo.

A total of 175 individuals from 45 unrelated families carrying seven different *TTR* pathogenic variants (p.Val50Met, p.Val142Ile, p.Ile127Val, p.Ile88Leu, p.Ala39Asp, p.Phe84Leu, p.Tyr98Phe) were identified. The most common variant was p.Val50Met (58.8%), followed by p.Val142Ile (29.7%) and p.Ile127Val (7.4%) (Figure 1A).

One hundred twenty‐two subjects were symptomatic (122/175, 69%), and 53 were asymptomatic carriers (Table 1). The self‐reported ethnicity was: 70.2% *Pardo* (admixed), 18.8% European ancestry (self‐reported as White), African ancestry 10.2% (self‐reported as Black), and 0.57% East‐Asian ancestry.

**TABLE 1:** Descriptive statistics.

Cohort description	
Patients, total (*N*, %)	175 (100%)
Sporadic cases (*N*, %)	22 (12.5%)
Familial cases (*N*, %)	153 (87.5%)
Symptomatic (*N*, %)	122 (69%)
Presymptomatic carriers (*N*, %)	53 (31%)
Median AOO (range)	50 (23–82)
Median age at diagnosis (range)	52 (23–84)

Genetic variability and presenting symptoms			
*TTR* variant^a^	V30M (p.Val50Met)	V122I (p.Val142Ile)	I107V (p.Ile127Val)
Total (%)	103 (58.8%)	52 (29.7%)	13 (7.4%)
Self‐reported ethnicity			
Pardo	57 (55.5%)	42 (80.7%)	9 (69%)
European ancestry	45 (43.6%)	0	0
African ancestry	0	10 (19.3%)	4 (31%)
East‐Asian	1 (0.9%)	0	0
Symptomatic *N* (%)	83 (80.5%)	25 (48%)	10 (77%)
Median AOO (range)	38.5 (23–73)	60 (35–72)	60 (48–70)
Median age at diagnosis (range)	41 (23–75)	60 (37–84)	61 (50–71)
Symptoms at onset			
Neuropathy	79 (95%)	11 (44%)	10 (100%)
Cardiopathy	0	11 (44%)	0
Mixed phenotype	4 (5%)	3 (12%)	0

Abbreviations: AOO, age of onset; *N*, number.

^a^ Only the three most common variants are reported in the table.

The participants were born in different regions of Brazil: São Paulo (50.9%), North/Northeast (19.1%), Central Region (17.9%), and South (12.1%). This observation is reflected in the district geographic distribution of TTR variants across the country. The V30M (p.Val50Met) and V122I (p.Val142Ile) variants are found all over the country, with a predominance of V30M (p.Val50Met) in São Paulo, as well as in the Central and Southern regions, while V122I (p.Val142Ile) is more common in the Northeast. I107V (p.Ile127Val) was only detected in Bahia state (Northeast) (Figure 1B).

### 3.2 Age of Onset

The median age of onset (AOO) in the overall cohort was 50 years (range: 23–82) (Table 1). In individuals harboring the V30M (p.Val50Met) mutation, AOO exhibited a broad distribution (median: 38.5 years; range: 23–73), with a characteristic bimodal pattern showing peaks at approximately 27 and 65 years, reflecting the coexistence of early‐onset (< 50 years old) and late‐onset (≥ 50 years) phenotypes within this subgroup (Figure 1C). Despite being less frequent, late‐onset V30M is not unusual in Brazil (37.4%).

Among patients carrying the V122I (p.Val142Ile) variant, the median AOO was 60 years (range: 35–72), and those with the I107V (p.Ile127Val) variant also exhibited a predominantly late‐onset disease (median: 60 years; range: 48–70) (Figure 1C).

Three V30M (p.Val50Met) unrelated families presented a presumable pattern of genetic anticipation, characterized by a reduction in AOO ranging from 8 to 36 years across generations (Family 1: I.1, II.3, II.5; Family 2: I.1 and II.1; Family 3: I.1 and II.1) (Figure S2).

### 3.3 Clinical Onset

In the entire cohort, neuropathy was the most frequent clinical feature at onset (86.1%), followed by cardiopathy (9.7%) and mixed phenotype (4.2%). V30M (p.Val50Met) variant was predominantly associated with neuropathic onset (79/83, 95%), mainly small fiber neuropathy (48/79, 60.7%). As previously described, while the V30M early‐onset cases start more often with small fiber neuropathy (44/52, 83%), there is a clear predominance of large fiber neuropathy among the late‐onset cases (23/31, 70.4%) (Figure 1D).

All individuals with the V107I (p.Ile127Val) variant presented with neuropathy at disease onset. In contrast, patients with the V122I (p.Val142Ile) variant exhibited the most diverse initial phenotypes, including cardiomyopathy (11/25, 44%), neuropathy (both small and large fiber) (11/25, 44%), and mixed presentations (3/25, 12%) (Figure 1D). Interestingly, neurological onset among V122I *Pardo* patients was more common than expected, including five cases presenting with early‐onset small fiber neuropathy (Table S1).

Concerning rarer variants, three individuals were identified with the I68L (p.Ile88Leu) variant: one presymptomatic, one with late‐onset cardiomyopathy, and one with early‐onset small fiber neuropathy. Two presymptomatic individuals carried the A19D (p.Ala39Asp) variant. A single patient with the T78P (p.Tyr98Phe) variant presented with late‐onset small fiber neuropathy, while one individual harboring the P64L (p.Phe84Leu) variant exhibited a late‐onset mixed phenotype. No patient in this cohort presented with renal, ocular, or other non‐neurological and non‐cardiological manifestations as the initial symptoms of ATTRv.

### 3.4 Factors Impacting the Age of Onset and Age at Diagnosis

Complementary investigations incorporated into the institutional diagnostic protocol facilitated the early identification of amyloidosis in selected cases. Skin biopsy was performed in 22 individuals presenting with paresthesia and dysesthesia despite normal nerve conduction studies, consistent with suspected small‐fiber neuropathy. Reduced intradermal fiber density was confirmed in 59% of cases (13/22), including five individuals carrying the V122I variant and eight with the V30M variant. Amyloid deposition was not assessed in these skin biopsies.

In addition, sural nerve biopsy was undertaken in five V30M cases, abdominal fat pad biopsy in two V30M cases, and endomyocardial biopsy in two V122I cases, with diagnostic confirmation achieved in all instances. Bone scintigraphy has been routinely available in our service since 2023. In this cohort, 66 symptomatic individuals were assessed using this technique, and 30% (20/66) demonstrated grade 2 or 3 uptake at initial evaluation, predominantly among those with the V122I variant (14/20, 70%). Notably, no cardiac uptake was observed in nine cases of early‐onset V30M.

According to literature [1, 2, 7], we next tested whether known modifying factors of disease onset—apart from the specific *TTR* variant—such as ethnicity, sex, family history, and close clinical monitoring apply to the Brazilian population (Table 2).

**TABLE 2:** Descriptive and inferential statistics.

Factors impacting AOO and diagnosis				
	Self‐reported ethnicity^a^			
	Pardo	White	Black	*p*
*N* (%)	87 (71%)	23 (18%)	12 (11%)	
Median AOO (range)	54 (23–75)	44 (24–82)	64 (50–77)	0.01^a^
Median age at diagnosis (range)	58 (23–77)	45 (25–84)	69 (53–80)	0.004

	Sex		*p*
	Male	Female	
*N* (%)	74 (53.7%)	48 (46.2%)	
Median AOO (range)	54 (23–75)	44 (27–82)	0.56
Median age at diagnosis (range)	55.5 (23–77)	45.5 (27–84)	0.73

	Family history		*p*
	Familial	Sporadic	
*N* (%)	100 (81.9%)	22 (18.1%)	
Median AOO (range)	45 (23–82)	65.5 (35–75)	0.0001
Median age at diagnosis (range)	46 (23–84)	66 (42–77)	0.0001

	Parent‐of‐origin		*p*
	Father	Mother	
*N* (%)	32 (43%)	42 (57%)	
Median AOO (range)	37 (23–69)	43.5 (24–82)	0.65
Median age at diagnosis (range)	39.5 (23–75)	45 (25–84)	0.90

Diagnosis during clinical monitoring			
	Yes	No	*p*
*N* (%)	22 (18.1%)	100 (81.9%)	
Median AOO (range)	44 (23–68)	53 (24–82)	0.16
Median age at diagnosis (range)	44.5 (23–68)	55 (25–84)	0.04^a^

*Note:* Diagnosis during clinical monitoring refers to presymptomatic carriers who started symptoms under medical follow‐up.

Abbreviations: AOO, age of onset; *N* = number.

^a^ East Asian (*n* = 1) was excluded.

In the entire cohort, patients with African heritage had a significantly later clinical onset and diagnosis than the *Pardo* and those with European ancestry. Sex and parent‐of‐origin did not show a statistically significant association with AOO or age at diagnosis. Conversely, the AOO of patients who reported a family history of amyloidosis was 20.5 years earlier than that of sporadic cases, reducing the age at diagnosis by 20 years, likely due to increased awareness in families and clinicians (Table 2).

Twenty‐two patients (with positive family history) were diagnosed during clinical monitoring of presymptomatic carriers, and 100 were referred to the center in the already symptomatic stage. Although the AOO between groups was different but not statistically significant, presymptomatic screening reduced age at diagnosis by 10.5 years (Table 2).

We next tested the relative contribution of specific *TTR* variants together with ethnicity and presence of family history on the age of onset. The overall regression model was statistically significant and explained 37% of the variance in the age of onset of the entire cohort (Table 3). The type of variant was a strong predictor, modulating on average 19 years of AOO, and patients with European ancestry started developing the disease roughly 10 years earlier, with a moderate statistical association between the type of variant and ethnicity (*χ* ^2^ = 34, *p* < 0.001, Cramer's V = 0.37). The family history also has an effect: those with a family member affected presented it around 7 years earlier than the sporadic ones (Table 3).

**TABLE 3:** Descriptive and inferential statistics.

Regression analysis				
	Entire cohort			*p*
	B (95% CI)	SE	β	
TTR variant				
Val142Ile (vs Val50Met)	19.1 (12.4, 25.7)	3.3	0.5	< 0.001
Ile127Val (vs Val50Met)	19.8 (10.2, 29.4)	4.8	0.3	< 0.001
Ethnicity				
European (vs Pardo)	−10.8 (−5, −16)	2.8	−0.3	< 0.001
African (vs Pardo)	3.6 (−7, 14)	5.3	0.5	0.5
Family history (Yes vs. No)	7.2 (0.01, 14.5)	3.6	0.1	0.04

V30M subgroup				
	B (95% CI)	SE	β	*p*
Ethnicity				
European (vs Pardo)	−9.5 (−14.8, −4.1)	2.6	−3.5	< 0.00
Family history (Yes vs. No)	6.9 (−1.7, 15.6)	4.3	0.1	1
Geographical region				0.1
Central (vs SP/South)	21 (14.4, 27.6)	3.3	0.5	< 0.001

*Note:* Regression analysis of entire cohort (Model F(6, 112) = 11.1, *p* < 0.001, *R* ^2^ = 0.37, adjusted *R* ^2^ = 0.34) and of V30M subgroup (Model F(5, 71) = 12.7, *p* < 0.001, *R* ^2^ = 0.47, adjusted *R* ^2^ = 0.43) were significant. Chi‐square test of independence showed a moderate association between ethnicity and type of variant (*χ* ^2^ = 34, *p* < 0.001, Cramer's V = 0.37) but no association between ethnicity and geographical region (*χ* ^2^ = 0.9, *p* = 0.4). Collinearity diagnostics indicated no major concerns; all VIF values were below 2.0, and tolerance values exceeded 0.5, suggesting that despite variables having a correlation, the degree of multicollinearity was not sufficient to bias the model. Β, standardized coefficient; CI, confidence interval; SE, standard error; SP, São Paulo.

Given the broad clinical variability, higher sample size, and wider geographical distribution, we conducted a specific analysis in the V30M subgroup by testing the effect of ethnicity and family history on AOO of V30M. As detailed data about the place of origin was available for all V30M cases, and *Pardo* and White V30M carriers were variably distributed across different regions, we decided to also include place of birth in the general model.

The regression model was statistically significant and explained 47% of the age of onset (Table 3). The place of birth was a highly strong predictor of AOO. Individuals from São Paulo and the South region (Paraná and Santa Catarina) of Brazil developed the disease on average 21 years earlier than those from the central region (Minas Gerais and Goiás) (Figure 1E,F). Self‐reported ethnicity showed a moderate modulating effect, and *Pardo* patients started the disease nearly 9 years later. Notably, there was no statistical association between birthplace and ethnicity (*χ* ^2^ = 0.9, *p* = 0.4). Family history was not statistically significant (Table 3).

## 4 Discussion

This study included participants with ATTRv from different regions in Brazil and diverse ethnic backgrounds. While V30M (p.Val50Met) remains the most common (58.9%), six additional variants were identified, reflecting a greater degree of genetic diversity and a wider geographic distribution than previously reported [8, 10].

For example, the I107V (p.Ile127Val) TTR variant, which is more commonly found in individuals of French origin, was identified exclusively in a specific region of central Bahia state among African and *Pardo* individuals. Despite the geographical isolation, the median age at onset (64 years) [11] is similar to that observed in France, with all cases presenting with neuropathy. We hypothesize that this pattern is likely due to a founder effect, as there are no known historical ties between this specific region and direct European ancestry.

Conversely, the predominance of V30M in São Paulo, Central, and Southern states can be attributed to higher levels of European migration over the past centuries, whereas the preponderance of V122I in the Northeast is likely explained by the strong influence of African ancestry in this region.

An interesting finding of our study is that in the Brazilian admixed population, 44% of V122I carriers presented with neuropathy in the absence of cardiac involvement. Despite this, the variant is typically associated with isolated late‐onset cardiomyopathy, with or without carpal tunnel syndrome [1, 2, 4]. However, increasing evidence suggests that up to 27%–38% of patients may develop peripheral neuropathy during the disease course [12, 13]. We acknowledge that since our center is a national referral site for peripheral neuropathies, we may have received a higher proportion of V122I cases with neuropathy than expected, which could have introduced a referral bias.

Brazil is considered endemic for the V30M variant, and previous studies from Rio de Janeiro state have predominantly reported early‐onset small fiber neuropathy, similar to V30M cases from Portugal [8]. In our cohort, however, we extended the V30M analysis to different geographic regions and ancestry. Interestingly, we observed that 37% of these individuals presented with late‐onset (> 50 years) neuropathy, more than previously reported [14].

Notably, late‐onset cases were more frequently observed in the Central Region (Goiás and Minas Gerais), whereas early‐onset cases were mostly detected in São Paulo and Southern states. This distribution suggests a possible contribution of environmental factors or distinct genetic backgrounds across the regions. Interestingly, we observed that individuals of mixed (*Pardo*) ethnicity tended to present at a later AOO compared to those of European ancestry. Although this may be partly explained by the enrichment of late‐onset cases carrying the V122I variant among *Pardo* patients, our analysis also suggests an independent effect of ancestry, regardless of the specific variant. Our observation supports previous studies suggesting that additional genetic modifiers or environmental factors may influence the age at onset and clinical features of ATTRv, warranting further investigation [7].

Our data showed that a positive family history leading to greater medical attention and the close monitoring of presymptomatic carriers substantially reduced the age at diagnosis, up to 10 years. This suggests that increasing medical awareness and improving access to care can enable earlier diagnosis and timely initiation of treatment. These findings are particularly relevant in non‐endemic regions, where late‐onset disease predominates [15, 16].

In conclusion, our findings expand the understanding of ATTRv in Brazil, revealing substantial genetic and phenotypic heterogeneity influenced by ancestry, geography, and healthcare access. Beyond confirming the predominance of V30M, we identified additional variants and novel clinical patterns, including a higher‐than‐expected frequency of neuropathy among V122I carriers and late‐onset presentations in V30M cases. These results underscore the importance of considering both genetic diversity and social determinants in the clinical evaluation and highlight the need for greater medical awareness and improved access to care to ensure timely diagnosis and treatment.

## Author Contributions

All authors contributed to the clinical evaluation and manuscript drafting.

## Funding

The authors have nothing to report.

## Disclosure

No figures were taken from any journals, websites, or other sources.

## Ethics Statement

This research was approved by the local and national ethics committee (CEP‐CONEP CAAE 74939423.4.0000.5440).

## Conflicts of Interest

The authors declare no conflicts of interest.

## Supporting information


**Figure S1:** Patient flow diagram showing the total number of patients screened, reasons for exclusion, and the final study population.
**Figure S2:** Pedigree with presumable anticipation phenomenon. AOO, age of onset; LFN, large fiber neuropathy; SFN AOO 35y, means the patient started with small fiber neuropathy symptoms at the age of 35; SFN, small fiber neuropathy.
**Table S1:** Clinical and paraclinical features of patients carrying V122I (p.Val142Ile) variant with neurological onset. CTS, carpal tunnel syndrome; IEFND, intraepidermal nerve fiber density; PREP, pain‐related evoked potentials; QST, quantitative sensory test; SNAP, sensory nerve action potential.

## Data Availability

The data that support the findings of this study are available from the corresponding author upon reasonable request.
