# Supplementary material for: Genetic and Clinical Spectrum of Hereditary Transthyretin Amyloidosis in Brazil
Source: J Peripher Nerv Syst. 2026 Jan 7;31(1):e70097. doi: 10.1111/jns.70097 (PMC12777902; doi:10.1111/jns.70097)
Supplement: Supplementary file 1 — Figure S1: Patient flow diagram showing the total number of patients screened, reasons for exclusion, and the final study population. Figure S2: Pedigree with presumable anticipation phenomenon. AOO, age of onset; LFN, large fiber neuropathy; SFN AOO 35y, means the patient started with small fiber neuropathy symptoms at the age of 35; SFN, small fiber neuropathy. Table S1: Clinical and paraclinical features of patients carrying V122I (p.Val142Ile) variant with neurological onset. CTS, carpal tunnel syndrome; IEFND, intraepidermal nerve fiber density; PREP, pain‐related evoked potentials; QST, quantitative sensory test; SNAP, sensory nerve action potential. [file JNS-31-0-s001.docx]

**Supplementary material**

Comorbidities representing risk factors for polyneuropathy were defined as follows: history of diabetes mellitus or use of insulin or oral antidiabetic drugs or abnormal fasting glucose tests; renal insufficiency: eGFR <60 ml/min/1.73 m2; abnormal thyroid function; vitamin B12 deficiency: serum vitamin B12<200 pg/ml; paraproteinemia: positive results in serum protein electrophoresis or immune fixation; diagnosed or suspected rheumatological diseases; positive tests for infectious diseases: HIV, syphilis, hepatitis or leprosy; historical of alcoholism and hypertriglyceridemia (above 200mg/dL).


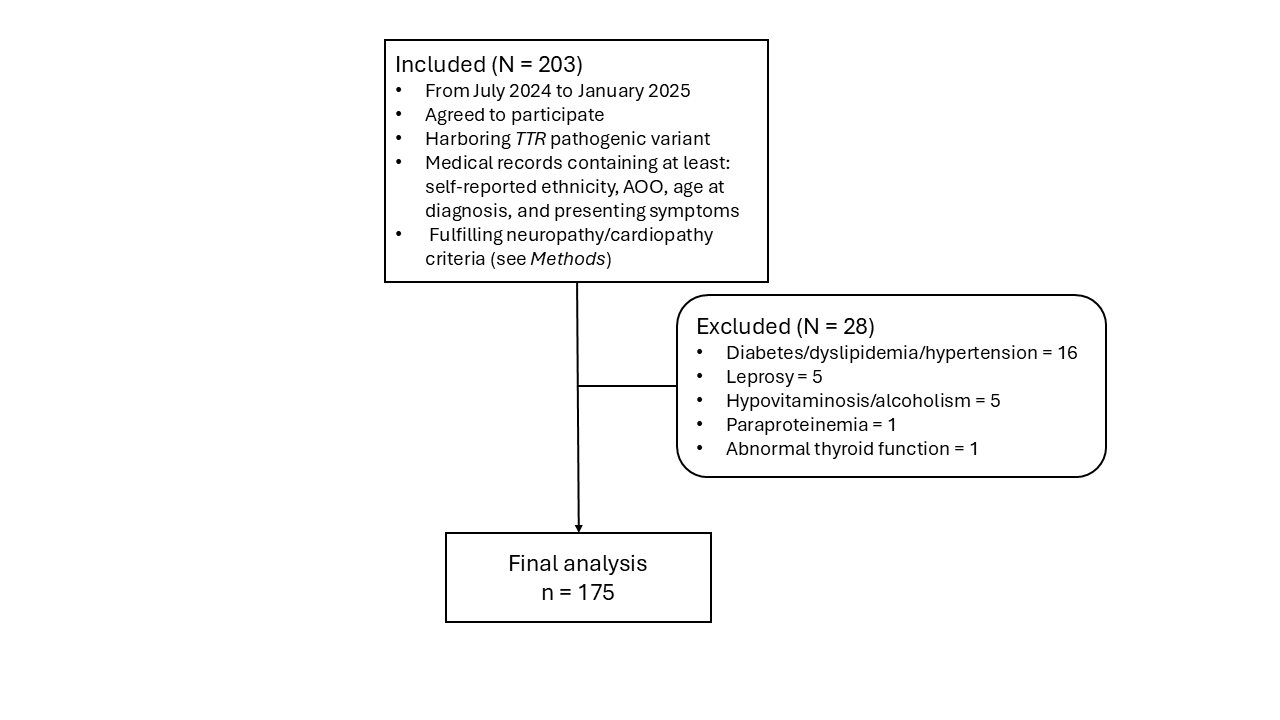


**Supplementary Figure 1**: Patient flow diagram showing the total number of patients screened, reasons for exclusion, and the final study population.


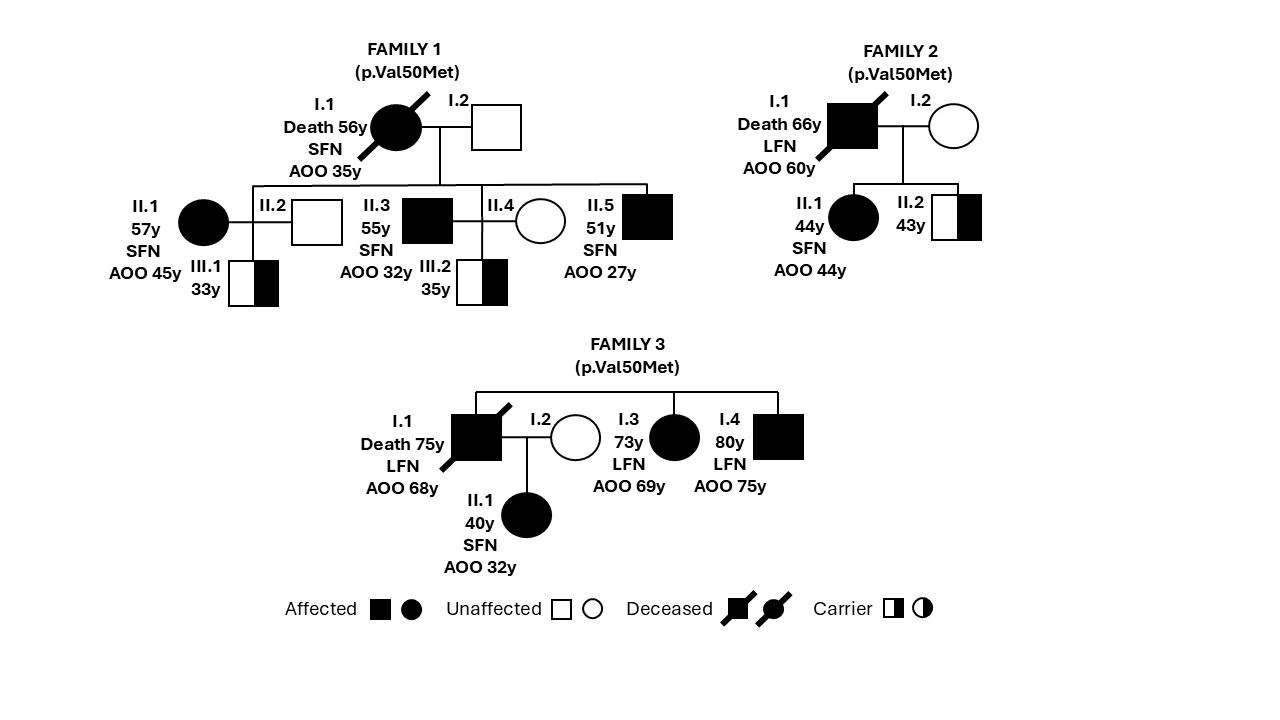


**Supplementary Figure 2**. Pedigree with presumable anticipation phenomenon. SFN = small fiber neuropathy, LFN = large fiber neuropathy, AOO = age of onset. SFN AOO 35y = means the patient started with small fiber neuropathy symptoms at the age of 35.

| **Patient number** | **Sex** | **Sporadic case** | **Parent-of-origin** | **Age of onset (years)** | **Symptoms at onset** | **Neurological examination** | **Complementary tests** | **Type of neuropathy**  **at onset** | **Age at clinical diagnosis**  **(years)** | **Diagnosis during presymptomatic screening** |
| --- | --- | --- | --- | --- | --- | --- | --- | --- | --- | --- |
| 1 | Female | No | Mother | 54 | Paresthesia and neuropathic pain | Length-dependent hypoesthesia | Abnormal PREP, QST, and Sudoscan.  Unilateral CTS. | Small fiber neuropathy | 54 | Yes |
| 2 | Male | No | Mother | 57 | Neuropathic pain | Length-dependent hypoesthesia | Abnormal Sudoscan, PREP, and sympathetic skin response.  Bilateral CTS. | Small fiber neuropathy | 59 | Yes |
| 3 | Male | No | Father | 37 | Diarrhea, dizziness, paresthesia, and erectile dysfunction | Length-dependent hypoesthesia and orthostatic hypotension | Abnormal sudoscan and sympathetic skin response. Bilateral CTS, unilateral cubital tunnel syndrome. | Small fiber neuropathy | 37 | Yes |
| 4 | Male | No | Father | 35 | Paresthesia and neuropathic pain | Length-dependent hypoesthesia | Abnormal PREP and Sudoscan.  Reduced IEFND in skin biopsy.  Bilateral CTS. | Small fiber neuropathy | 38 | No |
| 5 | Male | No | Mother | 48 | Paresthesia and erectile dysfunction. | Orthostatic hypotension | Abnormal sympathetic skin response and PREP. Bilateral CTS. | Small fiber neuropathy | 48 | No |
| 6 | Female | No | Mother | 41 | Neuropathic pain, diarrhea, and dry eyes | Weight loss | Abnormal PREP, QST, and Sudoscan. | Small fiber neuropathy | 41 | Yes |
| 7 | Male | No | Father | 55 | Neuropathic pain | Length-dependent hypoesthesia | Abnormal PREP and QST. | Small fiber neuropathy | 58 | No |
| 8 | Female | No | Father | 60 | Neuropathic pain and paresthesia | Normal | Abnormal Sudoscan and QST. | Small fiber neuropathy | 60 | No |
| 9 | Female | No | Unknown | 55 | Neuropathic pain | Normal | Reduced IEFND in skin biopsy.  Normal Sudoscan and QST. | Small fiber neuropathy | 55 | Yes |
| 10 | Male | Yes | - | 61 | Sensory loss | Impaired vibratory sensation | Reduced SNAP.  Bilateral CTS. | Large fiber neuropathy | 68 | No |
| 11 | Male | No | Unknown | 49 | Neuropathic pain, paresthesia, diarrhea and erectile dysfunction | Dry skin, patchy hair loss in legs/feet and orthostatic hypotension | Abnormal sympathetic skin response and Sudoscan.  Reduced IEFND in skin biopsy.  Bilateral CTS. | Small fiber neuropathy | 49 | Yes |

**Supplementary Table 1**. Clinical and paraclinical features of patients carrying V122I (p.Val142Ile) variant with neurological onset. PREP = Pain-Related Evoked Potentials. QST = Quantitative Sensory Test. CTS = Carpal Tunnel Syndrome. SNAP = Sensory Nerve Action Potential. IEFND = Intraepidermal Nerve Fiber Density.
